# Analysis of complete mitochondrial genome of *Ocypode cordimanus* (Latreille, 1818) (Decapoda, Ocypodidae)

**DOI:** 10.1080/23802359.2016.1168718

**Published:** 2016-06-20

**Authors:** Jin-Mo Sung, JeaHyun Lee, Seong-Geun Kim, Mustafa Zafer Karagozlu, Chang-Bae Kim

**Affiliations:** a Department of Life Science, Sangmyung University, Seoul, Korea;; b Bionics Co., Ltd, Seoul, Korea

**Keywords:** Complete mitochondrial genome, Decapoda, *Ocypode cordimanus*, Ocypodidae, phylogenetic tree

## Abstract

Complete mitochondrial genome of smooth-handed ghost crab *Ocypode cordimanus* (Latreille, 1818) has been sequenced and phylogenetic relationships evaluated due to mitochondrial protein coding genes. This is the second record of complete mitochondrial genome from the genus. The size of mitochondrial genome for *O. cordimanus* is 15,604 bp and the nucleotide distribution of the mitochondrial genome is 31.8% A, 21.8% C, 11.9% G and 34.5% T.

*Ocypode* is a genus belongs to Ocypodidae which lives in the sandy shores of tropical and subtropical regions. This genus is easily differentiated from similar genera due to their unique claws that have stridulating ridges on the palms (Sakai & Türkay 2013). There are 26 valid species in the genus (WoRMS 2016). Among them, only complete mitochondrial genome of *Ocypode ceratophthalmus* has been reported (Tan et al. 2014). In this study, complete mitochondrial genome of *Ocypode cordimanus* (Latreille 1818) evaluated and reported. Furthermore phylogenetic relationship of the species reconstructed based on amino acid sequences of mitochondrial genes.

The species have been collected from the sandy bottom of Osakura Island, Chook Lagoon, Federated States of Micronesia (7°28'40″N, 151°53'49″E) on 26 February 2015 and preserved in 97% ethanol. The specimen deposited in the Marine Biodiversity Institute of Korea (MABIK CR00235262). The size of mitochondrial genome for *O. cordimanus* (GenBank accession no. KT896743) is 15,604 bp which is slightly longer than *O. ceratophthalmus* (15,564 bp) and the AT content of the mitochondrial genome of the present species is 66.3% which is lower than that of *O. ceratophthalmus* (69.5%). The mitochondrial genome is composed of 13 protein-coding genes, two ribosomal RNA genes and 22 tRNA genes with 13 overlapping regions between 1 and 58 bp in length. Also, 14 intergenic sequences show length variation ranging from 1 to 46 bp and the largest intergenic sequence is located between *NAD4* and *NAD5* genes. The phylogenetic relationship shows that *O. cordimanus* is close to *O. ceratophthalmus* species and they belong to the monophyletic Ocypodoidae (Figure 1). Previous six nuclear and two mitochondrial genes-based molecular study (Tsang et al. 2014) showed that the Ocypodoidea has a sister group relationship with the Grapsoidea in superfamily level. Our data also support this relationship. This mitochondrial genome will provide molecular systematic usage for the reconstruction of Ocypodidae phylogeny.

**Figure 1.**
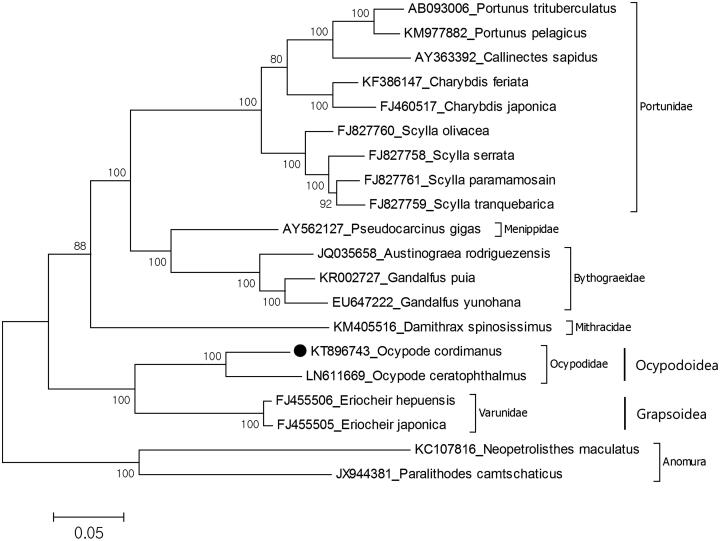
Molecular phylogeny of Ocypode cordimanus in the section Eubrachyura. The mitochondrial DNA was extracted from walking leg and purified. Purified libraries were profiled using the Agilent Bioanalyzer and sequenced with the Illumina MiSeq platform to yield 300 bp paired end reads. Mitochondrial genes were assembled and annotated by MITObim software (Hahn et al. 2013) and MITOS web server (Bernt et al. 2013). The annotation of mitochondrial genome sequences was refined by using Geneious software version 9.1.2 (http://www.geneious.com, Kearse et al. 2012). Comparison of mitochondrial genome control regions of two species shows that there are several common microsatellites in control regions of Ocypode such as (TA)3, (AT)3 and ATATAA. The most common motif is TA that seen 96 times in O. cordimanus while 116 times in O. ceratophthalmus. The phylogeny of O. cordimanus reconstructed with maximum likelihood statistical method by MEGA (Kumar et al. 1993). mtREV with Freqs (+F) model used for amino acid substitution and bootstrap method replicated 1000 times for the test of phylogeny. For reconstruction, the complete mitochondrial genomes of the Eubrachyura species were retrieved from the GenBank and amino acid sequences of all protein coding genes except ATP8 gene were used for analysis. The species belongs to the infraorder Anomura chosen as representative of outgroup.
